# Clinical outcomes and prediction nomogram model for postoperative hemoglobin < 80 g/L in patients following primary lumbar interbody fusion surgery

**DOI:** 10.1186/s13018-023-03766-w

**Published:** 2023-04-10

**Authors:** Xu Xiong, Jia-Ming Liu, Zi-Hao Liu, Jiang-Wei Chen, Zhi-Li Liu

**Affiliations:** 1 Medical Innovation Center, The First Affiliated Hospital of Nanchang University, No. 17 Yongwaizheng Street, Donghu District, Nanchang, 330006 Jiangxi Province People’s Republic of China; 2 Institute of Spine and Spinal Cord, Nanchang University, Nanchang, 330006 People’s Republic of China

**Keywords:** Lumbar interbody fusion, Hemoglobin, Predictors, Nomogram, Postoperative

## Abstract

**Objective:**

To analyze the association between different postoperative hemoglobin (Hb) levels and postoperative outcomes in patients who have undergone primary lumbar interbody fusion, and to investigate the risk factors and establish a predictive nomogram mode for postoperative Hb < 80 g/L.

**Methods:**

We retrospectively analyzed 726 cases who underwent primary lumbar interbody fusion surgery between January 2018 and December 2021in our hospital. All patients were divided into three groups according to the postoperative Hb levels (< 70 g/L, 70–79 g/L, ≥ 80 g/L). The postoperative outcomes among the three groups were compared, and the risk factors for postoperative Hb < 80 g/L were identified by univariate and multivariable logistic regression analysis. Based on these independent predictors, a nomogram model was developed. Predictive discriminative and accuracy ability of the predicting model was assessed using the concordance index (C-index) and calibration plot. Clinical application was validated using decision curve analysis. Internal validation was performed using the bootstrapping validation.

**Results:**

Patients with postoperative Hb < 80 g/L had higher rates of postoperative blood transfusion, a greater length of stay, higher rates of wound complications, and higher hospitalization costs than those with postoperative Hb ≥ 80 g/L. Preoperative Hb, preoperative platelets, fusion segments, body mass index, operation time, and intraoperative blood loss independently were associated with postoperative Hb < 80 g/L. Intraoperative blood salvage was found to be a negative predictor for postoperative Hb < 80 g/L (OR, 0.21 [95% CI 0.09–0.50]). The area under the curve of the nomogram model was 0.950. After internal validations, the C-index of the model was 0.939. The DCA and calibration curve suggested that the nomogram model had a good consistency and clinical utility.

**Conclusions:**

Postoperative Hb < 80 g/L in patients following primary lumbar interbody fusion surgery increased blood transfusions requirement and was independently associated with poor outcomes. A novel nomogram model was established and could conveniently predict the risk of postoperative Hb < 80 g/L in patients after this type of surgery.

## Background

Lumbar interbody fusion surgery has been considered the classic surgical treatment for lumbar degenerative diseases requiring spinal stabilization and fusion, such as lumbar spinal stenosis, disk herniation, and spondylolisthesis [1, 2]. This surgery may significantly relieve radicular symptoms and pain by removing nerve compression and providing spinal stabilization. However, the invasive nature of the procedure may adversely affect postoperative hemoglobin (Hb) levels, resulting in severe anemia [3]. Postoperative low Hb increases the need for blood transfusions and is associated with poor outcomes [4]. It is not clear what Hb threshold is the balance between the risk of transfusion and the risk of anemia. A postoperative Hb < 70 or < 80 with anemia symptoms is generally considered as the threshold for transfusion [5]. A restrictive transfusion strategy with a Hb threshold of 70–79 g/L is considered safe and feasible [6]. However, few studies have evaluated the relationship between clinical outcomes and different postoperative Hb levels.

A better understanding of the risk factors for postoperative low Hb will help reduce the incidence of postoperative anemia. Several studies have reported the risk factors of low Hb after surgery, including preoperative factors such as age, female gender, comorbidities, and preoperative Hb level, and intraoperative factors such as surgical duration, operative type, fusion segment, and intraoperative blood loss [7]. However, few studies have established a prediction model for postoperative low Hb in patients with primary lumbar interbody fusion.

Nomograms allow for the evaluation of many significant variables so that a certain clinical event of an individual patient can be accurately predicted. Nomograms may also provide visual results and make them easier to understand. Although some studies have developed a prediction model for blood transfusion during the perioperative period in lumbar interbody fusion, these studies did not include patients with low Hb levels without transfusions. Additionally, these patients usually have poor clinical outcomes [8]. Therefore, it is necessary to identify the risk factors and establish a prediction nomogram model for postoperative Hb < 80 g/L in patients following primary lumbar interbody fusion. We aimed to analyze the association between different postoperative Hb levels (< 70 g/L, 70–79 g/L, ≥ 80 g/L) and postoperative outcomes, investigate the risk factors, and establish a predictive nomogram model for postoperative Hb < 80 g/L in patients following primary lumbar interbody fusion.

## Materials and methods

### Patients

Following institutional review board approval, we conducted a retrospective review of the clinical data of patients who underwent transforaminal lumbar interbody fusion (TLIF) or posterior lumbar interbody fusion (PLIF) at our hospital between January 2018 and December 2021. We included: patients aged ≥ 18 years; who underwent primary lumbar interbody fusion; with complete clinical and laboratory data; and with a diagnosis of lumbar degenerative disease. All patients were divided into three groups (< 70 g/L, 70–79 g/L, ≥ 80 g/L) according to postoperative Hb.

### Data collection

The clinical data of patients were collected, including preoperative factors, intraoperative variables, and postoperative outcomes. Preoperative factors included: sex; age; body mass index (BMI); comorbidities (hypertension, hyperlipidemia, respiratory diseases, and diabetes); and laboratory data [Hb, platelets (PLT), prothrombin time, activated partial thromboplastin time, thrombin time, and fibrinogen]. A variety of intraoperative variables were assessed, including operative type (PLIF or TLIF), American Society of Anesthesiologists (ASA) class, operation time, intraoperative blood loss, intraoperative fusion segment, and intraoperative blood salvage. Postoperative variables included Hb level, length of hospitalization, wound complications, and hospitalization cost. All surgeries were performed by senior chief physicians. The postoperative Hb nadir was defined as the lowest Hb level measured during the hospital stay after surgery.

### Statistical analysis and nomogram model establishment

The statistical analysis was conducted using R (version 4.2.1) and SPSS software, version 26 (IBM Corp. Armonk, NY). Numbers and percentages were used to report descriptive statistics, and those for continuous variables are given as means ± SD. Relationships between different postoperative Hb levels and postoperative outcomes were assessed using linear regression analysis or logistics regression analysis. Potential factors were calculated using univariate analysis and categorical variables were analyzed using the Chi-square test, while continuous variables were analyzed using the Student’s t-test or the Mann–Whitney *U*-test. Then, we conducted a multivariate logistic regression to identify the independent predictors for Hb < 80 g/L. A *P* value < 0.05 (two-sided) was considered statistically significant.

Independent predictors of multivariate logistics regression analysis were used to establish a nomogram model. Subsequently, the predictive ability of the mode was assessed using the area under the curve (AUC) of the receiver operating characteristic (ROC). The prediction probability of the model was evaluated using the calibration curves and the nomogram discrimination performance was quantified by the C-index. For internal validation, the prediction model of the postoperative Hb < 80 g/L was subjected to 1000 bootstraps resamples, and the relatively corrected C-index was calculated. We performed a decision curve analysis (DCA) to assess the clinical benefit of the prediction model at different threshold probabilities.

## Results

### Patient population and postoperative outcomes

We included 726 patients undergoing primary lumbar interbody fusion surgery. Among these cases, 29 (4.0%) had a nadir level of postoperative Hb < 70 g/L, 54 (7.4%), 70–79 g/L; and 643 (88.6%), ≥ 80 g/L. For patients with postoperative Hb < 70 g/L, 79.3% of them (23 of 29) received allogeneic transfusions, while 53.7% of patients (29 of 54) received allogeneic transfusions in the 70–79 g/L Hb level group. Only five patients (0.8%) received allogeneic transfusions in the postoperative Hb ≥ 80 g/L group.

There were 7 cases (21.4%) with wound complication in postoperative Hb < 70 g/L group, 9 cases (16.7%) in the 70–79 g/L Hb level group, and 31 cases (4.8%) in Hb ≥ 80 g/L group. The average length of stay for the three groups (< 70 g/L, 70–79 g/L, ≥ 80 g/L) was 17.3 ± 3.6, 15.7 ± 3.9, and 12.8 ± 3.6 days. Mean hospitalization costs of the three groups were 8.0, 7.8, and 5.7 ten thousand RMB, respectively. Patients with postoperative Hb < 70 g/L or 70–79 g/L had a greater length of stay, higher rates of wound complications, and higher hospitalization costs than those with postoperative Hb ≥ 80 g/L. After adjusting for these potential confounders (age, sex, comorbidities, BMI, and fusion segments), postoperative Hb of < 80 g/L was independently associated with poor outcomes after lumbar interbody fusion surgery. The clinical outcomes were compared among the three groups (< 70 g/L, 70–79 g/L, ≥ 80 g/L) (Table 1).

**Table 1:** Association between postoperative Hb and outcomes

Outcomes	Postoperative Hb < 70 g/L	Postoperative Hb of 70–79 g/L	Postoperative Hb ≥ 80 g/L
Postoperative blood transfusion	23/29*^#^	29/54*	5/643
Length of stay (d)	17.3 ± 3.6*	15.7 ± 3.9*	12.8 ± 3.6
Wound complications	7/29*	9/54*	31/643
Hospitalization costs (ten thousand RMB)	8.0 ± 3.5*	7.8 ± 3.2*	5.7 ± 1.2

*Hb* hemoglobin

^*^Statistically significant differences from the Postoperative Hb ≥ 80 g/L are shown (*P* < 0.05)

^#^Statistical difference compared with Postoperative Hb of 70–79 g/L

### Independent risk factors for postoperative Hb nadir of < 80 g/L

The results of the univariate analysis to assess the predictors of postoperative Hb < 80 g/L are shown in Table 2. Nineteen variables were included and a statistical association with postoperative Hb nadir < 80 g/L was found in 11 variables. In multivariate logistics regression analysis, the backward-stepwise selection method was used to identify these predictive factors. These results demonstrated that BMI (OR, 1.18 [95% CI 1.05–1.33]), preoperative Hb (OR, 1.12 [95% CI 1.09–1.16]), preoperative PLTs (OR, 1.01 [95% CI 1.00–1.02]), three or more fusion segments (OR, compared with one segment 53.34 [95% CI 6.06–469.24]), operation time (OR, 1.01 [95% CI 1.00–1.02]), and intraoperative blood loss (OR, 1.00 [95% CI 1.00–1.02]) were independently associated with greater odds of postoperative Hb < 80 g/L. The intraoperative blood salvage (OR, 0.20 [95% CI 0.08–0.49]) was independently associated with lower odds of postoperative Hb of < 80 g/L (Table 3).

**Table 2:** Univariate analysis of postoperative Hb of < 80 g/L after primary lumbar interbody fusion

Variables	Hb < 80 g/L (*n* = 83)	Hb ≥ 80 g/L (*n* = 643)	*t*/*z*/*x*^2^	*P*
Age (years)	61.14 ± 10.61	54.97 ± 12.04	− 4.46	0.00
Sex			31.93	0.00
Male	12	303		
Female	71	340		
BMI	21.69 ± 3.19	23.75 ± 3.73	0.07	0.00
Comorbidities				
Hypertension	12/71	116/527	0.65	0.42
Hyperlipidemia	10/73	120/523	2.19	0.14
Respiratory diseases	6/77	22/621	2.87	0.09
Diabetes	7/76	40/603	0.60	0.44
Preoperative laboratory tests				
Hb	113.96 ± 15.57	134.57 ± 15.45	11.43	0.00
PLT	204.00 ± 61.72	226.36 ± 60.29	3.17	0.00
PT	10.86 ± 0.85	10.79 ± 0.80	− 0.71	0.48
APTT	26.81 ± 3.82	26.42 ± 3.43	− 0.97	0.33
TT	18.66 ± 2.04	18.31 ± 1.67	− 1.75	0.08
FIB	2.66 ± 0.70	2.71 ± 0.77	0.46	0.65
Operative type			7.76	0.01
PLIF	67	421		
TLIF	16	222		
ASA			9.54	0.00
≤ 2	198	445		
≥ 3	12	71		
Fusion segment			118.88	0.00
1	25	468		
2	41	170		
≥ 3	17	5		
Operation time (min)	218.61 ± 64.93	166.67 ± 42.46	− 9.77	0.00
Intraoperative blood loss (mL)	634.82 ± 370.46	323.75 ± 190.75	− 12.20	0.00
Intraoperative blood salvage	25/58	120/523	6.04	0.01

*Hb* hemoglobin, *BMI* body mass index, *PLT* platelet, *PT* prothrombin time, *APTT* activated partial thromboplastin time, *TT* thrombin time, *FIB* fibrinogen, *PLIF* posterior lumbar interbody fusion, *TLIF* transforaminal lumbar interbody fusion, *ASA* American Society of Anesthesiologist class

**Table 3:** Multivariable regression analysis of postoperative Hb of << 80 g/L after primary lumbar interbody fusion

Variables	OR	95%CI	*P*
Age	1.02	0.98–1.05	0.31
Sex	1.29	0.53–3.15	0.58
BMI	1.18	1.05–1.33	0.01
Preoperative Hb	1.12	1.09–1.16	0.00
Preoperative PLT	1.01	1.00–1.02	0.01
ASA	1.63	0.64–4.13	0.31
Operative type	0.49	0.21–1.14	0.10
Fusion Segment			
1	–	–	–
2	2.72	1.24–5.94	0.01
≥ 3	53.34	6.06–469.24	0.00
Operation time	1.01	1.00–1.02	0.01
Intraoperative blood loss	1.00	1.00–1.02	0.00
Intraoperative blood salvage	0.20	0.08–0.49	0.00

*OR* odds ratio, *CI* confidence interval, *BMI* body mass index, *Hb* hemoglobin, *PLT* platelet, *ASA* American Society of Anesthesiologist class

### Development and validation of the nomogram model

**Fig. 1:**
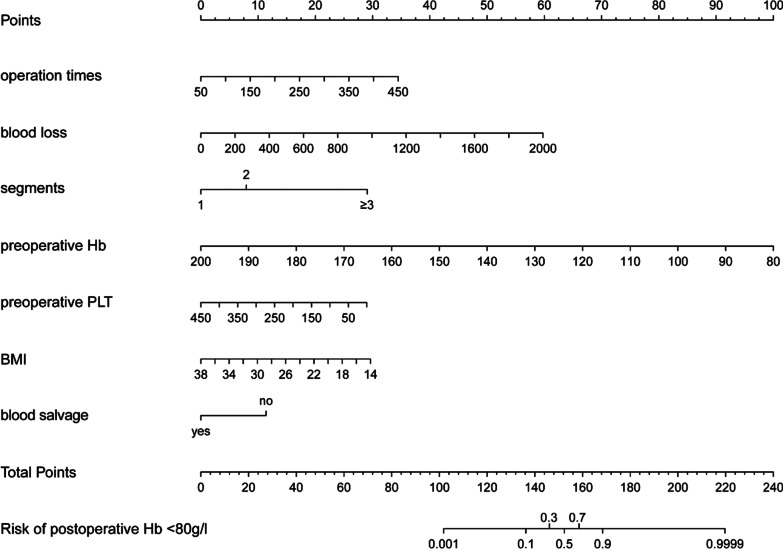
The developed postoperative Hb < 80 g/L nomogram. The nomogram was developed in the cohort with the use of preoperative Hb, preoperative PLT, operative time, intraoperative blood loss, fusion segments, and intraoperative blood salvage. *Hb* hemoglobin, *PLT* platelet, *BMI* body mass index

The nomogram model was established based on the seven independent risk factors identified by multivariate logistics regression analysis (Fig. 1). For example, if a patient underwent primary lumbar interbody fusion with two fusion segments, preoperative Hb of 120 g/L, PLT of 160 × 10^9^/L, BMI of 22 kg/m^2^, intraoperative blood salvage was utilized, operation duration was 200 min, and intraoperative blood loss was 400 mL, the total score based on the model was 7.5 + 66.5 + 33 + 22.5 + 0 + 22 + 12.5 = 164 points. The probability of postoperative Hb < 80 g/L was, therefore, up to 80% for this patient.

**Fig. 2:**
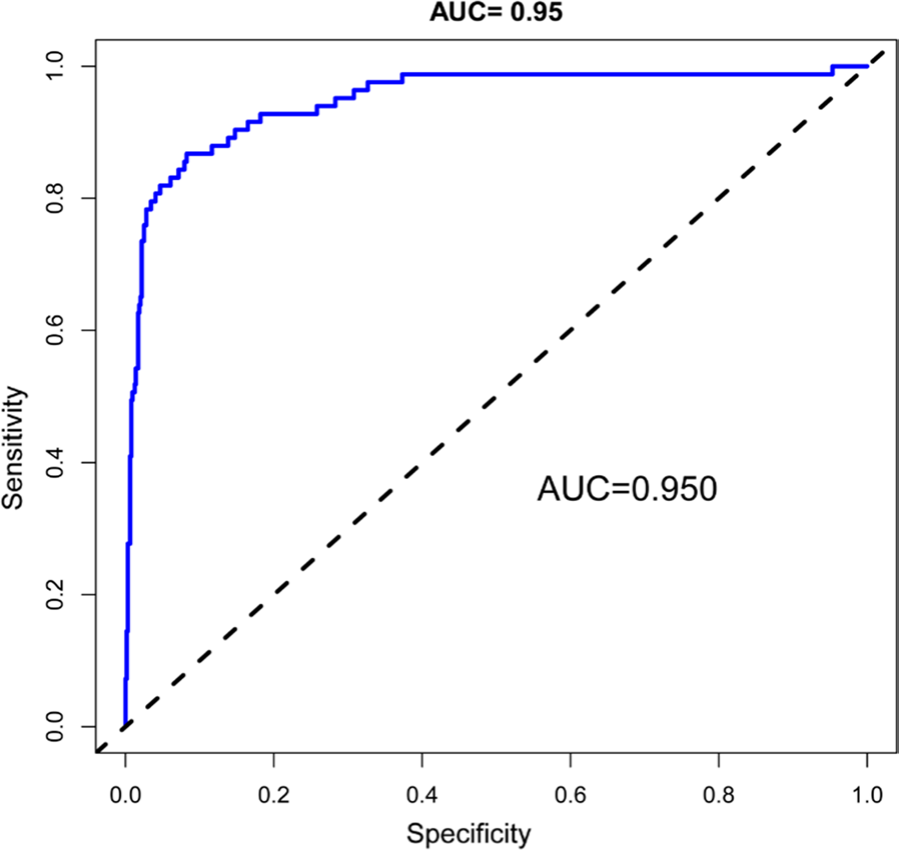
Receiver operating characteristic (ROC) curve to evaluate prediction accuracy. The AUC of the nomogram model was 0.950. *AUC* area under the curve

**Fig. 3:**
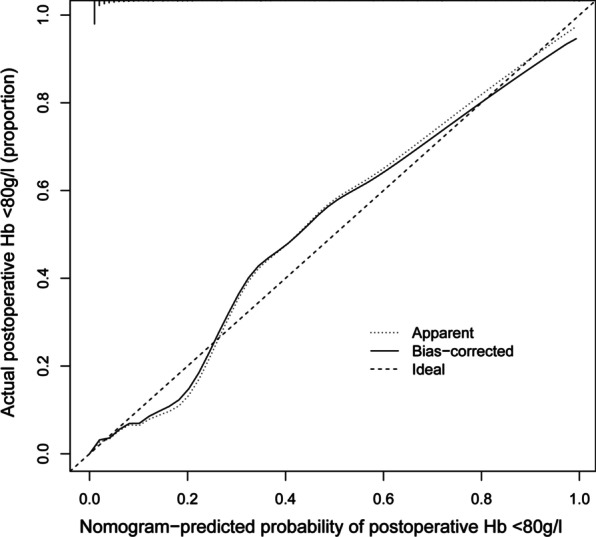
Calibration curves of the postoperative Hb < 80 g/L nomogram prediction in the cohort

**Fig. 4:**
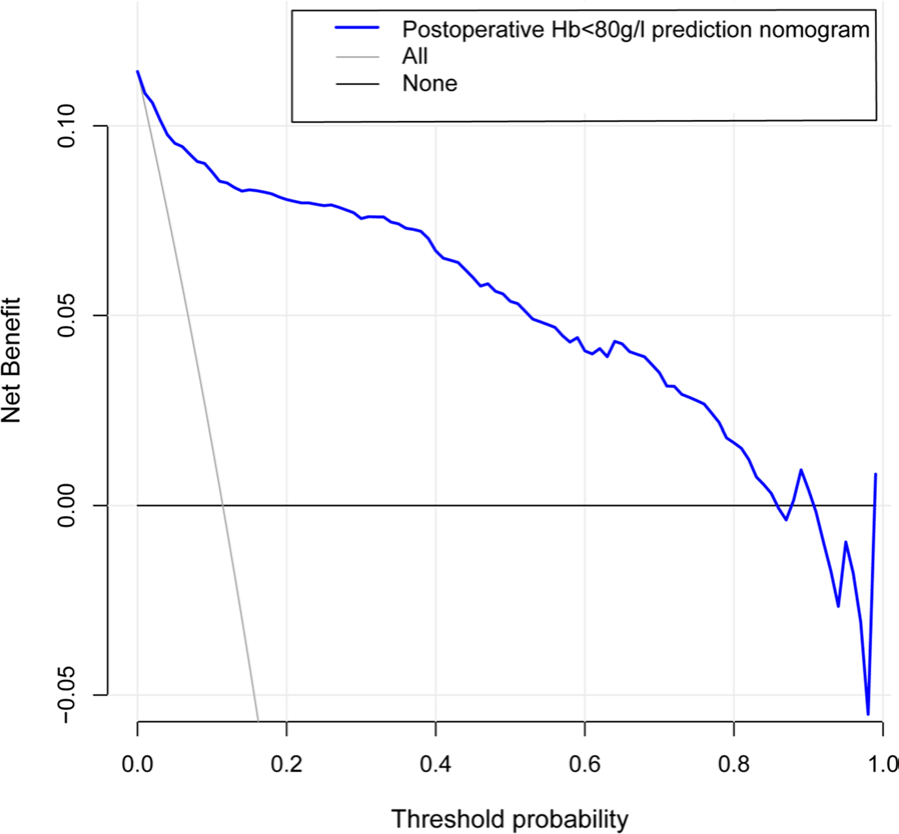
Decision curve analyses (DCA) for the postoperative Hb < 80 g/L nomogram

We evaluated the reliability of the nomogram model. The AUC for the predictive model was 0.950 (95% CI 0.923–0.978) (Fig. 2), indicating that the model had a strong discriminatory ability. A preoperative Hb level of 121.5 g/L maximized the specificity (79.3%) and sensitivity (71.1%) to predict postoperative Hb < 80 g/L. The C-index of the model using 726 patients was 0.950 (95% CI 0.922–0.977). Through bootstrap verification, the C-index was 0.939, which showed that the model had good discrimination. Additionally, the calibration curves were found to be in good agreement with actual measurements of postoperative Hb of < 80 g/L in primary lumbar interbody fusion patients (Fig. 3). The DCA showed that using this nomogram to predict a postoperative Hb of < 80 g/L had a net benefit across 0.01–0.84 threshold probabilities (Fig. 4).

## Discussion

Postoperative Hb < 80 g/L in patients following primary lumbar interbody fusion surgery was independently associated with poor outcomes. A novel nomogram model was established and could conveniently predict the risk of postoperative Hb < 80 g/L in these patient’s following surgery.

Patients undergoing lumbar interbody fusion usually suffer from postoperative anemia and require a blood transfusion; however, there has been controversy regarding the postoperative Hb thresholds for transfusion. Postoperative Hb < 70 g/L or < 80 g/L with anemia symptoms is generally considered as the threshold for transfusion [9]. The clinical practice guidelines from the American Association of Blood Banks strongly recommend a restrictive transfusion threshold of 80 g/L for patients undergoing orthopedic surgery [10]. Multiple randomized controlled trials have been conducted to examine the feasibility of postoperative Hb < 80 g/L as the threshold [11]. Chaudhry et al. [9] found that patients with postoperative Hb < 80 g/L were associated with poor outcomes, such as acute kidney injury, higher rates of readmissions, and longer hospital stays following total joint arthroplasty. In our study, patients with postoperative Hb levels < 70 g/L or 70–79 g/L after undergoing primary lumbar interbody fusion had higher rates of wound complications, longer hospital stays, and higher hospitalization costs than patients with postoperative Hb levels ≥ 80 g/L. However, there were no significant differences in the outcomes between patients with postoperative Hb levels < 70 g/L and Hb 70–79 g/L. Thus, we used postoperative Hb < 80 g/L as the threshold for blood transfusion attempted to identify the risk factors for these patients after fusion surgery.

Our findings suggest that lower preoperative Hb, lower preoperative PLT, lower BMI, longer operative times, greater intraoperative blood loss, and more fusion segments independently increased the odds of postoperative Hb < 80 g/L in patients who underwent primary lumbar interbody fusion. Most previous studies focus on the factors that may increase the risk of postoperative blood transfusions in orthopedic surgery patients. These factors are strongly associated with postoperative blood transfusions when there is low postoperative Hb. Similar to other studies, a recent systematic review found that low preoperative Hb significantly increased the risk of postoperative transfusion [12]. Kim et al. [13] reported that preoperative Hb level (OR 2.25, 95% CI [1.64–3.10]) was an independent predictor for transfusion after total knee arthroplasty. Similarly, Mozella et al. [14] reported that preoperative Hb levels ≤ 123 g/L is an independent predictor for postoperative blood transfusion after the same surgery. Similar to these studies, the optimal cutoff value of preoperative Hb in our study was 121.5 g/L. In multilevel thoracic and lumbar spine surgeries, Chow et al. [15] found that patients with low preoperative PLT required more blood transfusions, a phenomenon which we similarly observed. The possible reason for this may be that low preoperative PLTs may increase the probability of intraoperative bleeding. A study by Arishi et al. [16] found that lower BMI contributed to the risk of receiving postoperative blood transfusions. Frisch et al. [17] and Pedersen et al. [18] reported that blood transfusion rates were higher among patients with low BMIs than in patients with normal BMI after surgery. These findings were consistent with our study and may be related to the decreased BMI with the decrease in blood volume.

In our study, longer operative times, greater intraoperative blood loss, and more fusion segments were found to be the risk factors for postoperative Hb < 80 g/L in lumbar spinal fusion patients. These results were similar to previous studies. According to Manara et al. [19], revision hip surgery with longer operative times leads to greater blood loss and transfusion risks. Morcos et al. [20] found that the following factors significantly predict the need for postoperative blood transfusion in posterior lumbar spinal fusion: prolonged surgical time; open posterior approach; multilevel surgery; ASA ≥ 2; and sacrum involvement. Based on the study results of Aoude et al. [7], multilevel fusion and extended operative time independently predict the need for blood transfusions during lumbar fusion. Patients undergoing multilevel fusion posterior lumbar surgery with long operative duration and great intraoperative blood loss are more likely to experience postoperative low Hb.

Of particular interest, intraoperative blood salvage was found to be a negative predictor of postoperative Hb of < 80 g/L in our study. Intraoperative blood salvage is a process whereby blood is collected from the surgical site, filtered and washed to produce autologous blood for transfusion back into the patient. The main component of return transfusion is concentrated red cells, which could theoretically increase the Hb level. In a retrospective study of 50 patients, Chanda et al. [21] reported intraoperative blood salvage is safe and effective in patients undergoing thoracolumbar internal fixation and fusion. Although some studies reported the use of intraoperative blood salvage in orthopedic surgery could not serve to reduce the need for allogenic transfusion [22, 23], it plays a role in increasing postoperative Hb levels and reducing perioperative blood loss [24, 25].

In this study, we successfully established a prediction nomogram model to assess the risk of postoperative Hb < 80 g/L in patients undergoing primary lumbar interbody fusion. Seven predictors identified by multivariate regression analysis were included in the nomogram model. The internal validation showed that the model had good discrimination and calibration ability. With its large sample size, the high C-index (0.939) of interval validation demonstrated the usefulness and accuracy of this nomogram [26].

Wang et al. [27] developed a blood transfusion nomogram among 885 patients who underwent posterior lumbar spinal fusion, with an internally validated C-index of 0.898. Another study [8] described a blood transfusion nomogram model after PLIF and found AUC values of 0.881 and 0.890 for the training and validation sets, respectively. Compared to these models, our prediction model could give a better prediction for postoperative Hb < 80 g/L. For high-risk patients, it is necessary to take measures to increase preoperative Hb level and platelet counts, use intraoperative blood salvage, and take hemostasis measurements to reduce intraoperative blood loss.

This study also has some limitations: First, since this study was retrospective, there may be bias associated with data collection and incomplete data for some patients. A prospective study with a large sample size may make the conclusions of this study more convincing. Second, some patient variables, such as smoking and drinking, were not included in this study, which may also have led to a bias. Furthermore, different senior surgeons performed the surgery. Varying surgical skills may have resulted in different intraoperative blood loss results, and finally affected the results of the study. Last, the postoperative Hb nadir in this study was defined as the lowest Hb level measured during the hospital stay after surgery; the different test times for Hb may affect the results.

In conclusion, postoperative Hb < 80 g/L was associated with poor clinical outcomes in patients undergoing primary lumbar interbody fusion surgery. Preoperative Hb, preoperative PLTs, operative time, intraoperative blood loss, fusion segments, and intraoperative blood salvage were found to be significant predictors of postoperative Hb < 80 g/L in this study. Additionally, a novel nomogram model was established based on these seven predictors, which showed great discriminative ability and clinical practicability.

## Data Availability

The datasets generated and/or analyzed during the current study are not publicly available due data that are the private property of the authors prior to publication of the study may be compromised but are available from the corresponding author on reasonable request.

## Abbreviations

Hb: Hemoglobin
ASA: American Society of Anesthesiologists
AUC: Area under the curve
BMI: Body mass index
DCA: Decision curve analysis
PLIF: Posterior lumbar interbody fusion
PLTs: Platelets
ROC: Receiver operating characteristic
TLIF: Transforaminal lumbar interbody fusion
